# Comprehensive study of proteasome inhibitors against *Plasmodium falciparum *laboratory strains and field isolates from Gabon

**DOI:** 10.1186/1475-2875-7-187

**Published:** 2008-09-24

**Authors:** Andrea Kreidenweiss, Peter G Kremsner, Benjamin Mordmüller

**Affiliations:** 1 Medical Research Unit, Albert Schweitzer Hospital, BP118 Lambaréné, Gabon; 2 Department of Parasitology, University of Tübingen, Tübingen, Germany

## Abstract

**Background:**

The emergence and spread of *Plasmodium falciparum *resistance to almost all available antimalarial drugs necessitates the search for new chemotherapeutic compounds. The ubiquitin/proteasome system plays a major role in overall protein turnover, especially in fast dividing eukaryotic cells including plasmodia. Previous studies show that the 20S proteasome is expressed and catalytically active in plasmodia and treatment with proteasome inhibitors arrests parasite growth. This is the first comprehensive screening of proteasome inhibitors with different chemical modes of action against laboratory strains of *P. falciparum*. Subsequently, a selection of inhibitors was tested in field isolates from Lambaréné, Gabon.

**Methods:**

Epoxomicin, YU101, YU102, MG132, MG115, Z-L_3_-VS, Ada-Ahx_3_-L_3_-VS, lactacystin, bortezomib (Velcade^®^), gliotoxin, PR11 and PR39 were tested and compared to chloroquine- and artesunate-activities in a standardized *in vitro *drug susceptibility assay against *P. falciparum *laboratory strains 3D7, D10 and Dd2. Freshly obtained field isolates from Lambaréné, Gabon, were used to measure the activity of chloroquine, artesunate, epoxomicin, MG132, lactacystin and bortezomib. Parasite growth was detected through histidine-rich protein 2 (HRP2) production. Raw data were fitted by a four-parameter logistic model and individual inhibitory concentrations (50%, 90%, and 99%) were calculated.

**Results:**

Amongst all proteasome inhibitors tested, epoxomicin showed the highest activity in chloroquine-susceptible (IC50: 6.8 nM [3D7], 1.7 nM [D10]) and in chloroquine-resistant laboratory strains (IC50: 10.4 nM [Dd2]) as well as in field isolates (IC50: 8.5 nM). The comparator drug artesunate was even more active (IC50: 1.0 nM), whereas all strains were chloroquine-resistant (IC50: 113 nM).

**Conclusion:**

The peptide α',β'-epoxyketone epoxomicin is highly active against *P. falciparum *regardless the grade of the parasite's chloroquine susceptibility. Therefore, inhibition of the proteasome is a highly promising strategy to develop new antimalarials. Epoxomicin can serve as a standard to compare new inhibitors with species-specific activity.

## Background

Treatment and control of *Plasmodium falciparum *infections in highly endemic regions strongly rely on chemotherapy [1]. However, parasite resistance to existing antimalarials is spreading rapidly and might disseminate to artemisinins, the current mainstay of treatment against drug-resistant parasites in the near future. Therefore, the development of new treatment strategies is of great importance.

The ubiquitin/proteasome system regulates the turnover of most proteins in eukaryotic cells and hence, plays an essential role in controlling protein quality, cell proliferation, cell death, and signal transduction. In *P. falciparum *protein quality control is of particular importance because: i) erythrocytic stage parasites have a high replication rate, ii) plasmodial proteins are large in size, iii) low complexity regions are abundant between and within globular domains, and iv) proteins are stressed by increased temperature in the host (fever). Those features are important challenges to the protein folding and degradation machinery. To avoid lethal accumulation of non-functional or misfolded proteins, protein quality needs to be tightly controlled. Previous studies show that in plasmodia two T1 threonine peptidase systems are present. The 20S proteasome is enzymatically active and expressed throughout the live cycle, whereas PfhslV is expressed in late stages of development [2], only.

Several studies investigated a single T1 threonine peptidase inhibitor (herein after referred to as proteasome inhibitor) to show its potential as a drug development candidate [2-5] but a comprehensive study on available classes of inhibitors is not available. Simultaneous testing of multiple inhibitor classes reveals the most potent inhibitor class amongst all inhibitors tested under identical assay conditions and indicates interactions between individual compounds. If a known antimalarial drug is included, the potency of the inhibitor can be directly evaluated in relation to the activity of the comparator drug and possible pharmacodynamic interactions can be revealed.

So far, all studies with proteasome inhibitors were done in laboratory isolates only. It is important to assess the activity of a drug candidate against fresh *P. falciparum *isolates from the field. These parasites are genotypically and phenotypically different from laboratory adapted strains and are very diverse in their genetic background. Differences in the range of activities between laboratory and field isolates cannot be predicted and a high variance in drug-activities in field isolates can indicate natural heterogeneity and a propensity to develop resistance against the candidate.

Several classes of proteasome inhibitors have been identified and a number of inhibitors have entered clinical trials. Previous studies proved proteasome inhibitors of various classes to influence growth of *P. falciparum *[2-5]. Here, representatives of peptide and non-peptide proteasome inhibitors classes were screened for their potency against *P. falciparum *laboratory strains. The most promising agents were further investigated in fresh isolates from malaria patients in Lambaréné, Gabon.

## Materials and methods

### Parasites

Screening of proteasome inhibitors were conducted with *P. falciparum *strains 3D7, D10 and Dd2. Parasites were obtained from MR4 (ATCC, USA). Subsequently, a selection of inhibitors was tested in field isolates from Gabon. Therefore, blood samples (n = 81) of children with uncomplicated malaria were collected at the Medical Research Unit of the Albert Schweitzer Hospital, Lambaréné, Gabon. Inclusion criteria were: I) *P. falciparum*-monoinfection (parasitemia between 10^3 ^and 1.2*10^5 ^parasites/μl blood), II) age between one and fifteen years, and III) no intake of antimalarial drugs for at least one month. A venous blood sample (0.5 ml) was drawn, washed once with parasite culture medium and applied immediately in the drug susceptibility assay. Informed consent and assent were obtained from the patients and/or their parents before collection of blood. The study was approved by the ethics committee of the International Foundation of the Albert Schweitzer Hospital in Lambaréné.

Consistency of the results over the study period was tested by correlation analysis of admission number and IC50s against all drugs to exclude temporal trends and degradation of study compounds (endoperoxides such as artesunate are particularly prone to decomposition).

### Reagents

Epoxomicin (MW: 555), YU101 (MW: 635, Calbiochem), YU102 (MW: 501), MG132 (MW: 476), MG115 (MW: 461), Z-L_3_-VS (MW: 551), Ada-Ahx_3_-L_3_-VS (MW: 936) and gliotoxin (MW: 326) were resuspended in dimethyl sulfoxide at a stock concentration of 10 mM each. PR11 (MW: 1464) and PR39 (MW: 4721) were dissolved in sterile PBS to a final concentration of 1 mM. Bortezomib (Velcade^®^, MW: 384, Millennium) was purchased as a 9 mM solution for intravenous injection. Lactacystin (MW: 376) and chloroquine diphosphate (MW: 515, Sigma-Aldrich) were prepared in double distilled water at 10 mM each. Artesunate (MW: 384) was dissolved in 70% ethanol. The maximum concentration of either solvent per well did not exceed 0.0007%. Ninety-six well flat-bottomed plates (Becton Dickinson) were pre-dosed with compounds using the following range of test concentrations: 1.6 – 100 nmol/L for epoxomicin, 7.8 – 500 nmol/L for YU101, MG132, and MG115, 0.2 – 10 mmol/L for YU102, lactacystin, gliotoxin, and PR11, 4.1 – 3,000 nmol/L for bortezomib, 0.6 – 10,000 nmol/L for Z-L_3_-VS, 1.4 – 1,000 nmol/L for Ada-Ahx_3_-L_3_-VS, 0.02 – 1 mmol/L for PR39, 1.4 – 1,000 nmol/L for chloroquine diphosphate and 0.1 – 52 nmol/L for artesunate. Control wells were pre-dosed with parasite culture medium only. All compounds were purchased from Biomol if not otherwise stated.

### Drug susceptibility assay

Drug susceptibility assays with laboratory parasite strains as well as with field isolates were done according to published standard procedures [6], with minor modifications. Parasitaemia was adjusted to 0.05% with non-infected O^+^-erythrocytes. Erythrocytes were resuspended in parasite culture medium (RPMI 1640, 25 mM HEPES, 2 mM L-glutamine, 50 μg/ml gentamicin, and 0.5% albumax) to a hematocrit of 1.5%. Two hundred microlitres of blood-medium-mixture were added to each well of the pre-dosed 96-well test plates. The plates were incubated for 72 hours at 37°C in a candle jar. To assess successful *in vitro *growth of field isolates, a thick blood smear of one control-well (untreated) was done after 26 hours and a similar sample was frozen at the same time to calculate background histidine rich protein-2 (HRP2)-production. Parasite culture was judged successful when at least 20% parasites matured to schizonts at the 26 hours time point. After 72 hours plates were freeze-thawed twice. Parasite growth, calculated from HRP2-levels, was measured with a commercially available enzyme linked immunosorbent assay (Malaria Ag CELISA, Cellabs, Australia), according to the manufacturers' specifications.

Number of field isolates tested per drug depended on inhibitory concentration immediately calculated after accomplishment of drug susceptibility assay. Highly active drugs (50% inhibitory concentration [IC50] < 100 nM) were tested at least 20 times and drugs with modest activity (IC50 > 100 nM) 10 times or more.

### Parasite growth in erythrocyte pre-incubated with proteasome inhibitors

Red blood cells were resuspended in parasite culture medium to a hematocrit of 5% and incubated for three hours in 37°C with proteasome inhibitors at a concentration well above their respective IC99: epoxomicin (50.0 nM), YU101 (0.5 μM), MG132 (5.0 μM), MG115 (0.5 μM), Z-L_3_-VS (1.0 μM) and Ada-Ahx_3_-L_3_-VS (50.0 nM). After extensive washings pre-treated erythrocytes were used for culture with ring stages of 3D7 and Dd2. Parasite culture and assessment of growth was determined as it is described above. Untreated erythrocytes served as control for growth. All experiments were done in triplicates.

### Statistical analysis

Inhibitory concentrations were determined by non-linear regression analysis of log-dose-response curves using R v2.3.1. (The R Foundation for Statistical Computing). Median IC50, 90 and 99 were calculated. Correlations between proteasome inhibitors and chloroquine were assessed by Pearson correlation coefficient (r) of logarithmically transformed IC50 values. Level of significance was set at both-sided p = 0.05 for all tests. All other statistical analysis was done with JMP v5.0.1.2 (SAS Institute).

## Results

Peptide proteasome inhibitors, a β-lactone and a group of allosterically acting inhibitors were screened for their *in vitro *activity against *P. falciparum *strains 3D7 and D10 (both CQ-susceptible), and against Dd2 (CQ-resistant). Subsequently, a selection of inhibitors was tested in field isolates from Gabon.

**Figure 1:**
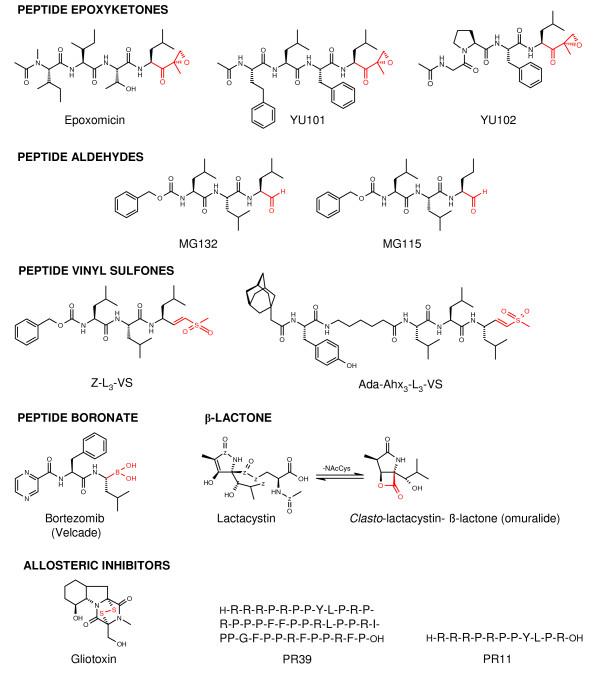
**Chemical structure of proteasome inhibitors**. Pharmacophores are shown in red. PR39 and PR11: amino acid sequence is shown; binding sites are so far unknown.

Peptide proteasome inhibitors are small molecules based on a short oligopeptide with a carboxy-terminal reactive group, such as α',β'-epoxyketone, aldehyde, vinyl sulfone or boronic acid (Figure 1). The short peptide sequence is mediating proteasome subunit specificity and the pharmacophore covalently binds reversible (aldehyde, boronic acid) or irreversible (epoxyketones, vinyl sulfone) to the active site amino-terminal threonine of the proteasome subunits. *Clasto*-lactocystin β-lactone, the active intermediate of lactacystin, is a cyclic compound and irreversibly inhibits proteasome function via an ester bonding. Gliotoxin and the proline and arginine-rich peptides PR39 and PR11, respectively, are structurally different and all inhibit the proteasome by allosteric binding [7,8].

### Screening of proteasome inhibitors against *P. falciparum *laboratory strains

Inhibitory concentrations of artesunate and chloroquine were determined in 3D7, D10 and Dd2 and served as a benchmark for activity and non-activity, respectively.

Amongst the peptide α',β'-epoxyketone inhibitors the antiparasitic potency of epoxomicin and its synthetic analogue YU101 is very high (Table 1). Only epoxomicin is in the range of artesunate's antiparasitic activity. YU102, demonstrating a high value for inhibition of the caspase-like activity compared to YU101, did not block growth of any parasite strain tested.

**Table 1:** Screening of 50% inhibitory concentrations of proteasome inhibitors in *P. falciparum *laboratory strains 3D7, D10 and Dd2

	**IC50 (nmol/L)^a^**		
	
**Test compounds**	**3D7**	**D10**	**Dd2**
1. Peptide proteasome inhibitors			
Epoxomicin	6.8	1.7	10.4
YU101	24.5	17.0	13.8
YU102	1740	4000	3006
MG115	97.5	50.0	44.9
MG132	22.4	33.2	17.3
Z-L_3_-VS	16.4	^n.d.^	4.9
Ada-Ahx_3_-L_3_-VS	300	^n.d.^	210
Bortezomib	252	127	561
			
2. β-lactone			
Lactacystin	1490	1211	2553
			
3. Allosteric inhibitors			
Gliotoxin	2171	^n.d.^	1165
PR11	^n.i.^	^n.d.^	^n.i.^
PR39	^n.i.^	^n.d.^	^n.i.^
			
4. Comparator drugs			
Artesunate	0.5	2.5	0.9
Chloroquine	8.3	5.0	176

^**a **^Values are expressed as mean IC_50 _from two separate determinations against chloroquine-susceptible 3D7 and D10 and chloroquine-resistant Dd2 clones.

^**n.d. **^assay not done

^**n.i. **^no growth inhibition detectable

Though both peptide aldehydes MG132 and MG115 are less active than epoxomicin, MG132 shows an inhibitory profile in the lower nanomolar range and is superior over MG115 in both CQ-susceptible and CQ-resistant parasites.

The tested inhibitors of the peptide vinyl sulfone class show a wide range of activities. Whereas the potency of Z-L_3_-VS is in the low nanomolar range, the inhibitory concentration of Ada-Ahx_3_-L_3_-VS is 19 times higher in 3D7 and 42 times in Dd2. Bortezomib, a peptide boronate, is the only proteasome inhibitor in clinical use so far. In contrast to multiple myeloma treatment [9], its activity in *P. falciparum *laboratory strains was low. The non-peptide based inhibitor lactacystin did not demonstrate inhibition of parasites' growth at low concentration. Amongst the group of allosterical inhibitors, gliotoxin demonstrated some modest activity whereas no inhibition was detectable for the peptide antibiotic PR39 and its derivative PR11 in the concentration range tested.

### Growth of 3D7 and Dd2 in pre-treated erythrocytes

To exclude growth inhibition of parasites due to inhibited host cell proteasomes uninfected erythrocytes were incubated with epoxomicin, YU101, MG132, MG115, Z-L_3_-VS and Ada-Ahx_3_-L_3_-VS, respectively. Pre-treated red blood cells were used for cultivation of 3D7 and Dd2. No growth inhibition was observed after 72 hours.

### *In vitro *activity of selected proteasome inhibitors in *P. falciparum *field isolates from Gabon

Based on the screening in laboratory strains of *P. falciparum *the following proteasome inhibitors were selected for testing in fresh isolates obtained from malaria patients from Lambaréné, Gabon: the most effective peptide α',β'-epoxyketone inhibitor epoxomicin and MG132. Even though bortezomib and lactacystin showed only minor antimalarial activity in laboratory strains, they were chosen. Bortezomib is the only proteasome inhibitor which is in use in humans. Lactacystin has been thoroughly tested in *P. falciparum *in previous studies and served as a benchmark for antiplasmodial activity of proteasome inhibitors tested in this study. Testing of YU101 and Z-L_3_-VS was abandoned because of difficulties in compound availability during study conduct.

**Figure 2:**
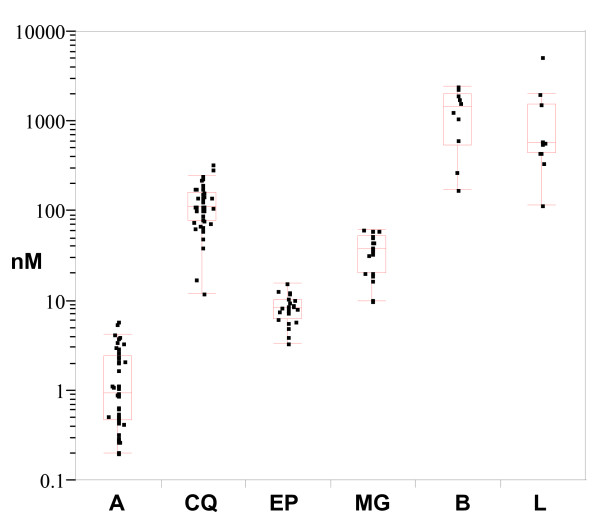
**Distribution of individual 50% inhibitory concentrations**. Distribution of single IC50s of artesunate (A), chloroquine (CQ), epoxomicin (Ep), MG132 (MG), bortezomib (B) and lactacystin (L) in *P. falciparum *field isolates. Outlier box plots show median IC50 and ends of the box are the 25% and 75% quantiles, respectively. The whiskers extend from the ends of the box to the outermost data point that falls within the distances computed. Dots outside whiskers are possible outlier IC50s. Note: concentration (nM) is shown in logarithmic scale.

*In vitro *drug susceptibility was tested in 22 (epoxomicin and MG132) and 11 isolates (bortezomib and lactacystin), respectively. All isolates were chloroquine resistant whereas artesunate was highly active (Table 2). Epoxomicin showed the highest molecular activity with a range of activities in individual samples that were remarkably narrow (Figure 2). Potency of MG132 proved to be as high as in laboratory strains. Activity of bortezomib and lactacystin in field isolates was very low with a wide range of activity in the individual samples. No proteasome inhibitor showed any significant cross correlations neither with chloroquine- nor with artesunate-activities.

**Table 2:** Median inhibitory concentrations of study drugs in *P. falciparum *field isolates of Gabon^a^

**Drug**	**no**.	**IC50**	**IC90**	**IC99**
Epoxomicin	22	**8.50 **(3.36 – 15.9)	**12.4 **(4.30 – 44.8)	**20.5 **(4.96 – 89.5)
MG132	22	**38.2 **(10.0 – 63.9)	**99.5 **(43.4 – 216)	**206 **(77.3 – 454)
Bortezomib	11	**1452 **(175 – 2477)	**2500 **(312 – 2871)	**2911 **(348 – 2990)
Lactacystin	11	**588 **(118 – 5318)	**2015 **(169 – 6289)	**3318 **(254 – 9278)
Artesunate	43	**0.96 **(0.20 – 5.95)	**2.47 **(0.33 – 29.9)	**5.76 **(0.57 – 49.1)
Chloroquine^b^	43	**113 **(12.4 – 332)	**241 **(20.1 – 737)	**544 **(40.2 – 967)

^a ^Results are shown in nM (median [range])

^b ^All isolates are beyond the threshold level of resistance (1 μM corresponds to 30 nM in our assay conditions)

## Discussion

In view of increasing levels of *P. falciparum *resistance to antimalarial drugs currently in use, there is an urgent need to develop new treatment strategies. Proteasome inhibitors are in the focus of drug development for various diseases and candidates have already entered clinical trials [10]. In plasmodia several studies show the potential of a single proteasome inhibitor as a drug development candidate [2-5]. Even though different growth detection methods give highly comparable results, assay parameters, mainly the initial parasitaemia used in the drug susceptibility assay, affects IC50 values [11]. Thus comparison of inhibitor activities between different studies is problematic, particularly if no comparator drug is tested in parallel. Here, the first comprehensive study on proteasome inhibitors against *P. falciparum *is provided. The most promising inhibitor amongst all classes tested was identified and its activities were compared with artesunate, one of the most active antimalarial compounds known. Moreover, differences in activities between the inhibitor classes are not biased by assay parameters because all compounds were tested in parallel and under exactly the same assay conditions.

The knowledge of activity of antimalarial drug candidates against field isolates is critical to estimate their potential in the drug development process. Freshly isolated parasites are heterogeneous in their genetic background and represent the local plasmodial population, including pre-existing mutations that account for drug resistance, whereas laboratory isolates are oligo- or monoclonal and are heavily selected for *in vitro *growth. It is important to establish the variability of efficacy of antimalarials (as it is seen in the range of IC50 values) against parasites with a diverse genetic background. The discrepancies seen between assays carried out on (fresh) patient isolates and on laboratory adapted strains cannot be predicted. Bortezomib showed a remarkably lower activity against *P. falciparum *field isolates then it was seen in laboratory strains. Furthermore, the variability in susceptibilities of patient isolates to one proteasome inhibitor class can be compared to the variability of susceptibilities to another compound. These comparisons can point to potential mechanisms of drug action as well as drug resistance. Resistance against chloroquine is highly prevalent in the study area and exclusion of cross-resistance with chloroquine is of major concern.

Peptide a',β'-epoxyketones are the most potent and specific proteasomal inhibitor class in mammalian cells so far [12]. In *P. falciparum *the peptide epoxyketone epoxomicin, an actinomycetes metabolite, showed the highest antimalarial activity in laboratory strains as well as in field isolates, independent of the chloroquine-susceptibility of the parasites. The narrow range of activity in the individual samples argues for a low potential of resistance-development. Interestingly, caspase-like subunit specific YU102 [13] did not affect parasite growth, whereas YU101 [14], a chymotrypsin-like specific inhibitor, inhibited parasites in the low nanomolar range. YU101 and YU102 are synthetic derivates and their peptide modifications were designed to target only one proteasomal proteolytic subunit with a high degree of specificity and potency. Even though epoxomicin is not specific for the plasmodial proteasome, YU-compounds show that it is principally possible to design epoxyketones towards subunit specific inhibition. If more is known about plasmodial proteasome particularities, like their low complexity regions in their active subunits [2], engineering of plasmodia specific epoxyketones should be feasible. The peptide vinyl sulfone analogue of MG132, Z-L_3_-VS, was considerable more potent in *P. falciparum *then Ada-Ahx_3_-L_3_-VS, a vinyl sulfone with an amino-terminal extension. This was surprising because in mammalian proteasomes the extended peptide vinyl sulfones were shown to be superior in potency over their shorter counterparts [15]. Presumably, the amino-terminal extension of Ada-Ahx_3_-L_3_-VS is hampering the molecule's permeability to the three membranes of *P. falciparum *infected erythrocytes. Although MG132 showed promising antimalarial activity regardless of the chloroquine susceptibility of the parasites, a major drawback of peptide aldehydes is their lack of proteasomal specificity. The proline and arginine-rich peptides PR39 and PR11 regulate the proteasome activity in a substrate-specific manner. They are unique in that they bind allosterically to the α subunits of the proteasome and change the shape of the proteasome. Only the degradation of a small subset of proteins is affected and thus antimicrobial peptides are less toxic than conventional competitive proteasome inhibitors [8]. In plasmodia no activity could be observed, however, inhibition of purified plasmodial proteasomes was not investigated. Although lactacystin and gliotoxin have already been shown active against *P. falciparum *[3,5], in the light of other proteasome inhibitor classes and comparator drugs their activity was of minor significance.

Currently, proteasome inhibitors are intensely investigated for their antimicrobial activity by several academic groups and companies. We hope to set a standard for pre-clinical *in vitro *studies of antimalarial inhibitors and look forward to the further development of lead compounds into clinical trials.

## Conclusion

Epoxomicin is highly active against *P. falciparum *and shows no signs of cross-resistance with the comparator drugs or any other proteasome inhibitor in an area with high-grade chloroquine resistance. Epoxomicin is a benchmark for the development of highly active, species-specific proteasome inhibitors. Additionally, epoxomicin is a valuable tool in plasmodial proteasome investigations.

## Competing interests

The authors declare that they have no competing interests.

## Authors' contributions

AK collected the samples, accomplished all drug sensitivity assays, performed the statistical analysis and drafted the manuscript. BM designed and coordinated the study as well as the statistical analysis. PK and BM revised the manuscript. All authors read and approved the final manuscript.
